# Open-Source Photometric System for Enzymatic Nitrate Quantification

**DOI:** 10.1371/journal.pone.0134989

**Published:** 2015-08-05

**Authors:** B. T. Wittbrodt, D. A. Squires, J. Walbeck, E. Campbell, W. H. Campbell, J. M. Pearce

**Affiliations:** 1 Department of Materials Science & Engineering, Michigan Technological University, Houghton, MI, United States of America; 2 The Nitrate Elimination Company, Inc., Lake Linden, MI, United States of America; 3 Department of Electrical & Computer Engineering, Michigan Technological University, Houghton, MI, United States of America; CAS, CHINA

## Abstract

Nitrate, the most oxidized form of nitrogen, is regulated to protect people and animals from harmful levels as there is a large over abundance due to anthropogenic factors. Widespread field testing for nitrate could begin to address the nitrate pollution problem, however, the Cadmium Reduction Method, the leading certified method to detect and quantify nitrate, demands the use of a toxic heavy metal. An alternative, the recently proposed Environmental Protection Agency Nitrate Reductase Nitrate-Nitrogen Analysis Method, eliminates this problem but requires an expensive proprietary spectrophotometer. The development of an inexpensive portable, handheld photometer will greatly expedite field nitrate analysis to combat pollution. To accomplish this goal, a methodology for the design, development, and technical validation of an improved open-source water testing platform capable of performing Nitrate Reductase Nitrate-Nitrogen Analysis Method. This approach is evaluated for its potential to i) eliminate the need for toxic chemicals in water testing for nitrate and nitrite, ii) reduce the cost of equipment to perform this method for measurement for water quality, and iii) make the method easier to carryout in the field. The device is able to perform as well as commercial proprietary systems for less than 15% of the cost for materials. This allows for greater access to the technology and the new, safer nitrate testing technique.

## Introduction

Human activities, such as treatment of sewage and other wastewaters, industrial and automotive combustion, and intensive agricultural crop fertilization and livestock production, have resulted in an overabundance of nutrients in groundwater and virtually all waters of the earth [1–3]. Among all environmental problems in the U.S., excess nutrients in American water systems is probably the most costly, widespread, and difficult to solve problem [4]. At harmful levels, nitrate, the most oxidized form of nitrogen, is a threat to human and animal health both directly and indirectly. Nitrate and nitrite in drinking water are regulated by the Safe Drinking Water Act [5]; and in wastewater by the Clean Water Act [6]. These government regulations help to protect people against harmful levels of nitrate and nitrite, which can result in methemoglobinemia, a deadly blood disorder [7]. Nitrate pollution can also lead to harmful bacterial and algal growth in wells, lakes, and estuaries [8]. Among the certified methods to detect and quantify nitrate and nitrite is the cadmium reduction method, which uses a toxic heavy metal (EPA Method 353.2 [9]). An alternative green method for nitrate and nitrite analysis is enzymatic reduction using nitrate reductase [10–12], which has recently been proposed by the US Environmental Protection Agency (EPA) as Alternate Test Procedure for determining nitrate [13]. The enzymatic nitrate analysis method has been verified to yield results equivalent to the accepted EPA certified methods [10–12]. Clearly, one way to begin to address the nitrate pollution problem is to promote facile field testing for nitrate. Since the Nitrate Reductase Nitrate-Nitrogen Analysis Method requires a spectrophotometer [10–12], the development of an inexpensive portable, handheld photometer will greatly expedite field nitrate analysis to facilitate detection of nitrate pollution.

Free and open source hardware (FOSH) and software (FOSS) design methodologies have been proven to be a useful for radically reducing the cost of scientific equipment while maintaining accuracy and precision [14–24]. The concept allows for digital reproduction of vetted designs for scientific analysis that are shared freely after the original design for approximately the cost of the materials [16,25]. Both the prototyping and distributed manufacturing of such scientific FOSH with a RepRap (self-**Rep**licating **Rap**id prototyper) [26–28] has been found to be useful for generating large-scale value in the scientific community [17,25,29–32]. Normally this method decreases the costs between a factor of 10 and 100 from commercially available technologies [17], which makes the tools accessible even in developing world contexts [33]. With the explosive growth of the consumer level 3-D printing industry largely predicated on RepRap technology [34] the scaling of any technical solution is substantial and growing rapidly.

Specifically related to nitrate testing, a RepRap 3-D printable free and open-source water testing platform has been developed capable of colorimetry and nephelometry [35,36]. This paper expands that work by providing a methodology for the design, development, and technical validation of an improved Open-Source water testing platform capable of performing the recently proposed EPA Nitrate Reductase Nitrate-Nitrogen Analysis Method. This approach is evaluated for its potential to i) eliminate the need for toxic chemicals in water testing for nitrate and nitrite, ii) reduce the cost of equipment to perform this method for measurement for water quality, and iii) make the method easier to carryout in the field. The results are discussed to provide conclusions about the future of nitrate and nitrite testing in both developed and developing regions.

## Materials and Methods

The low-cost water testing platform has been designed in OpenSCAD [37], an open-source, script based, 3-D modeling program and optimized to be manufactured using consumer level 3-D printers using polylactic acid (PLA) and thermoplastic elastomer (TPE) filaments. All the components of the design are thus parametric and capable of future customization. For example, the case has been designed to accommodate a modular cuvette holder that can be replaced with a holder of nearly any geometry to expand the capabilities of the device. Design elements allow the case to be assembled with zero added hardware and only three parts need to be printed. All parts are printed without support and can fit on the build plate of nearly all typical RepRap 3-D printers. An optional TPE case is included for improved hand feel and to allow some shock protection for use in the field in case of an accidental dropping. The designs were printed with a Lulzbot Taz 4, RepRap derived open-source 3-D printer (Aleph Objects).

**Fig 1:**
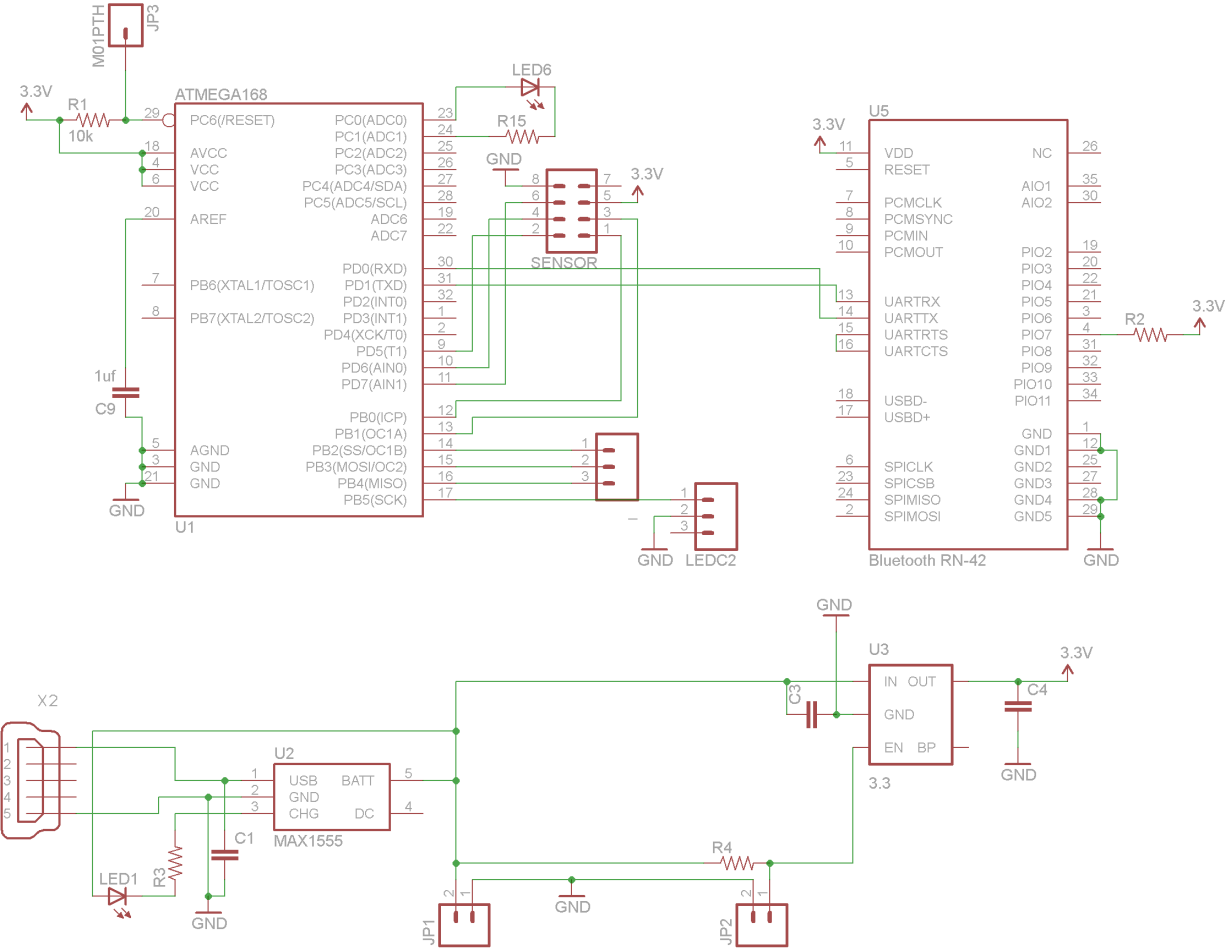
Schematic for open-source electronics 1) the main ATMega board, 2) Bluetooth module, and 3) Power.

**Fig 2:**
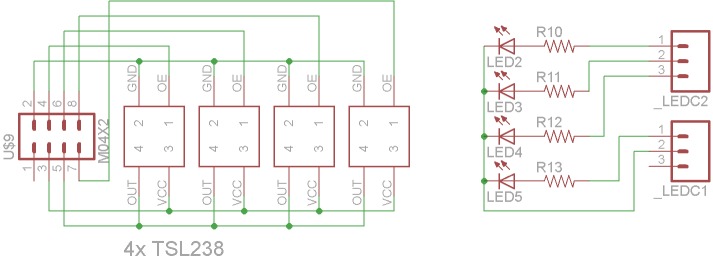
Schematic for 1) the sensor board and 2) LED board.

The open-source electronics for the device are based off of the open-source Arduino microcontroller [38] using an inexpensive ATMega 168 chip and have been designed to communicate over Bluetooth to the user’s mobile device using the Roving Networks RN-42 Bluetooth module [39]. Power is supplied to the device with a 500 mAh lithium ion battery with the voltage stepped down from 3.7V to 3.3V [40]. Fig 1 shows the electronic schematic for the main ATMega board, the Bluetooth module, and the electric power and Fig 2 shows the electronic schematics for the sensors and LED array, respectively.

The nitrate testing platform was calibrated and tested using 1000 ppm nitrate-N Certified Nitrate Standard (Fisher Scientific Co.) and with the manual enzymatic nitrate analysis method [10]. The nitrate standards are prepared from the Certified Nitrate Standard by dilution with nitrate-free deionized water to the following concentrations: 0.5, 1.0, 2.0, 2.5, 5.0, 7.5, and 10.0 ppm nitrate-N.

Manual nitrate analysis with NECi “Open-Source” Enzymatic Nitrate Analysis Method for Water is carried out by reconstituting freeze-dried recombinant NADH:nitrate reductase (AtNaR2, EC 1.7.1.1) in 25 mM potassium phosphate, 0.1 mM Na_2_EDTA, pH 7.5, at 22–25°C, to a final concentration of 1 unit per mL [10]. A total of 3 units of AtNaR2 are needed to analyze approximately 50 water samples and nitrate standards. The unit of enzyme activity is defined as the amount of AtNaR2 catalyzing conversion of 1 μmol nitrate to nitrite per min at pH 7.5 and 30°C. Using the reconstituted AtNaR2, the Nitrate Analysis Reagent is prepared by bringing the reconstituted AtNaR2 solution (3 units) to 54 mL by mixing with additional phosphate buffer, and the addition of 1.0 mL of 2.82 mM NADH solution, prepared in deionized water. Final volume of the Nitrate Analysis Reagent = 55 mL. It should be noted that this reagent is stable for about 1 hour and should be used immediately after the NADH is added.

The enzymatic reduction step in the nitrate analysis method is carried out by adding 50 μL of aqueous sample or nitrate standard to 1.0 mL of nitrate analysis reagent, mixing completely with a test tube mixer, and incubating the mixture at room temperature for 20 min. The nitrate in the standards and samples of unknowns is completely reduced to nitrite in this time period [9–11]. Reagent blanks are also prepared by replacing the water sample in the above step with 50 μL of deionized water.

The color development step, basically the Griess Reaction, is carried out by addition of 100 μL of sulfanilamide reagent (10 g per L of ~1.2 N HCl), with vigorous mixing and a 2 min reaction time, which is followed by addition of 100 μL N-(1-naphthyl)ethylenediamine dihydrochloride reagent (1.0 g per L of deionized water) and reacting mixture at room temperature, for at least 5 min. A longer reaction time is not harmful as long as the absorbance of the samples are read within 1 hour. The final mixture is transferred to a 1.5 mL plastic cuvette for reading in the photometer. Absorbance at 540 nm is read for each sample and nitrate standard using the open-source photometer, which actually calculates ppm nitrate-N from the absorbance using the internal standard curve in the smartphone app software. If the samples are suspected to contain nitrite and the user wishes to determine nitrite, this is simply done by adding 50 μL of sample to 1.0 mL of phosphate buffer or deionized water and reacting with the color reagents. After the color develops it is read with the photometer which will be reported by the smartphone app as ppm nitrite-N using the internal standard curve. The user also has the choice of displaying the results in units used in California, Europe, and the World Health Organization (WHO), which are ppm Nitrate and ppm Nitrite.

A cost comparison is conducted for the photometer hardware using the bill of materials for self-assembly of the open-source photometer and comparing to the retail cost of current equivalent photometers.

## Results

**Fig 3:**
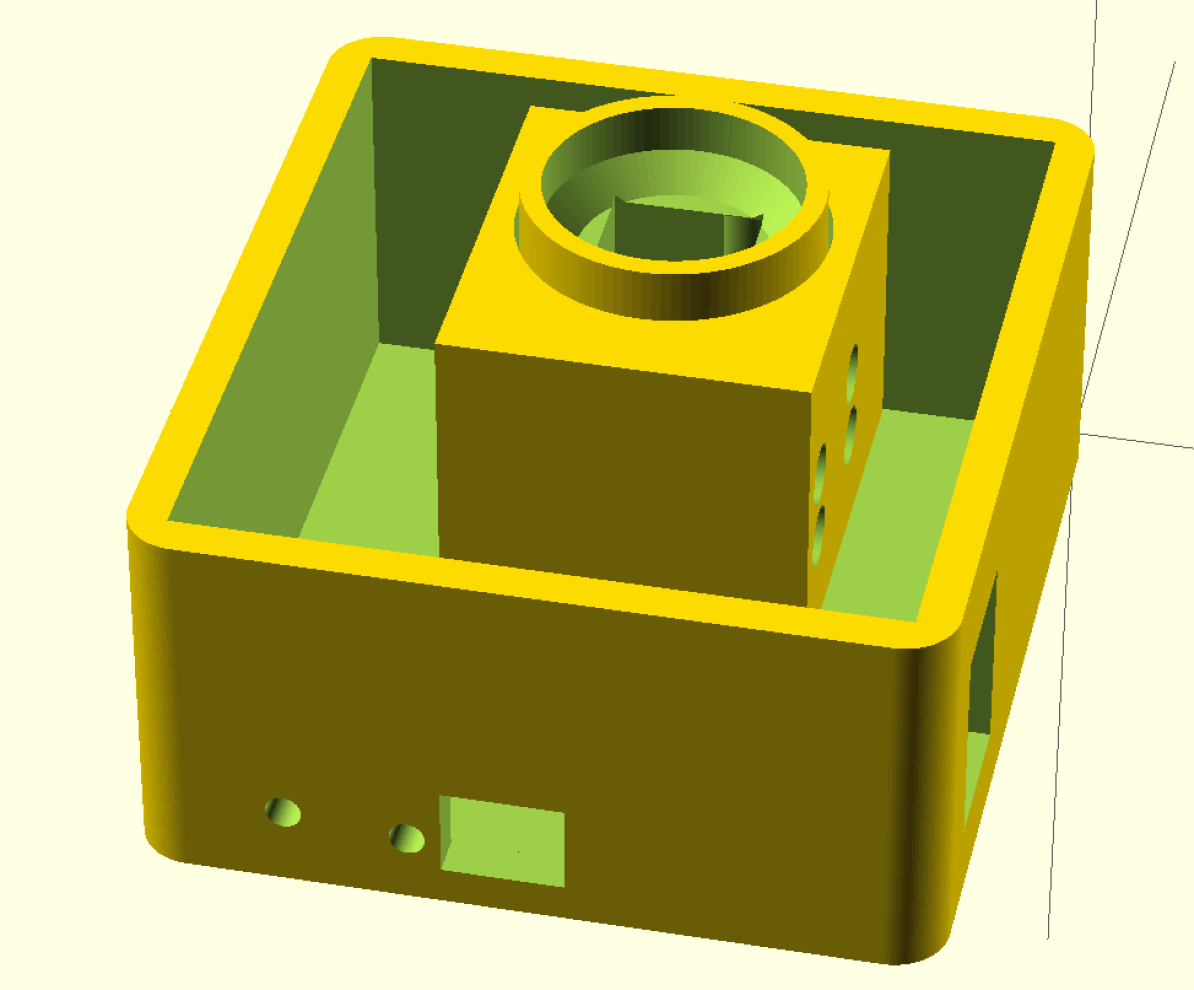
OpenSCAD screenshot of main photometer case.

The final design of the photometer case features modules that allow easy modification of individual parts of the photometer. This allows the user to easily change the analysis receptacle to accept different geometries and sizes of testing media. Additionally, all pieces of the case, bottom, cuvette holder, and top, can be generated within the single OpenSCAD file (see S1 Appendix). The composite of the bottom and cuvette holder modules are shown in Fig 3 within the OpenSCAD interface.

**Fig 4:**
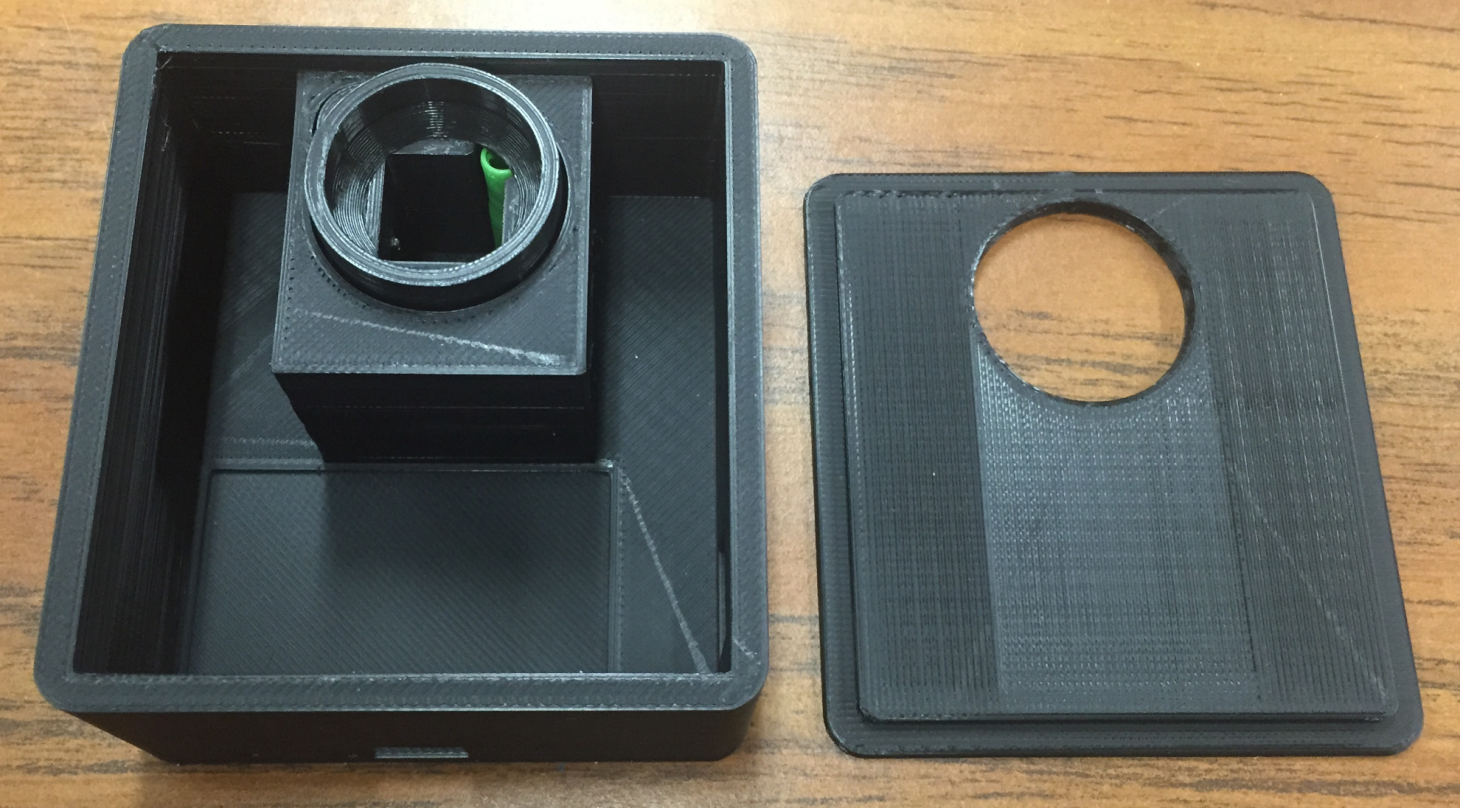
Photometer case with top removed.

**Fig 5:**
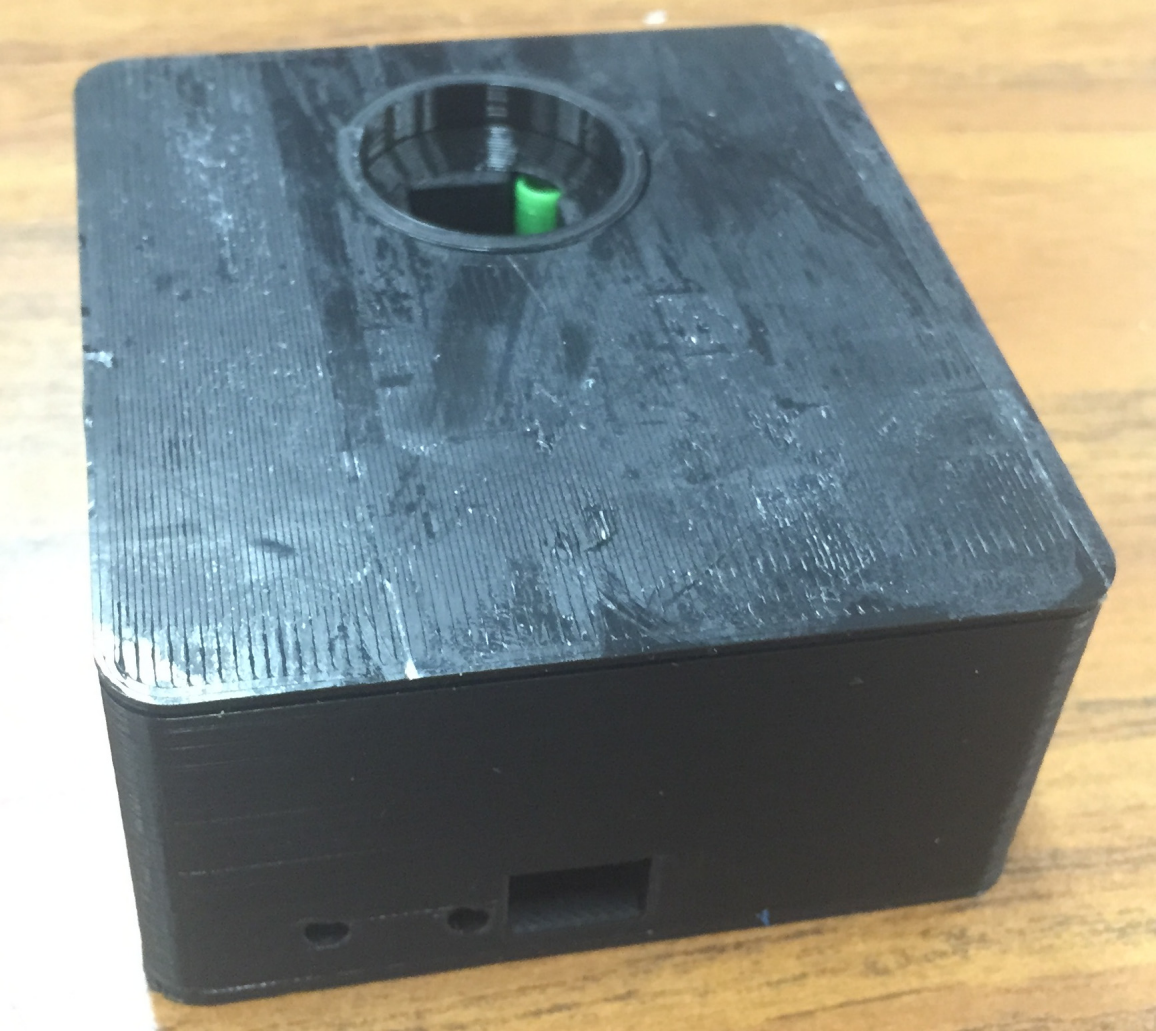
Assembled photometer case.

**Fig 6:**
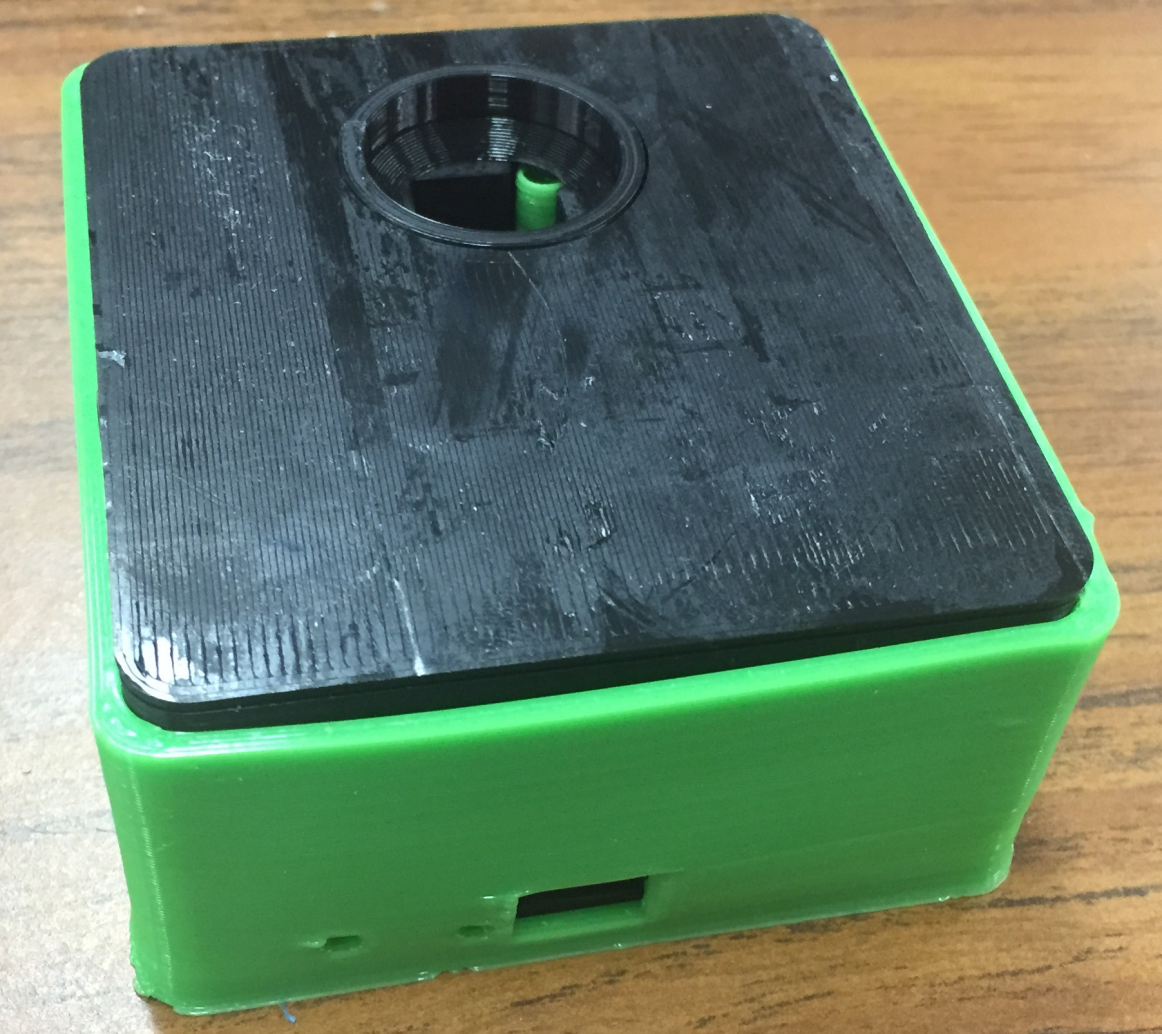
Photometer case with TPE protective outer case.

**Fig 7:**
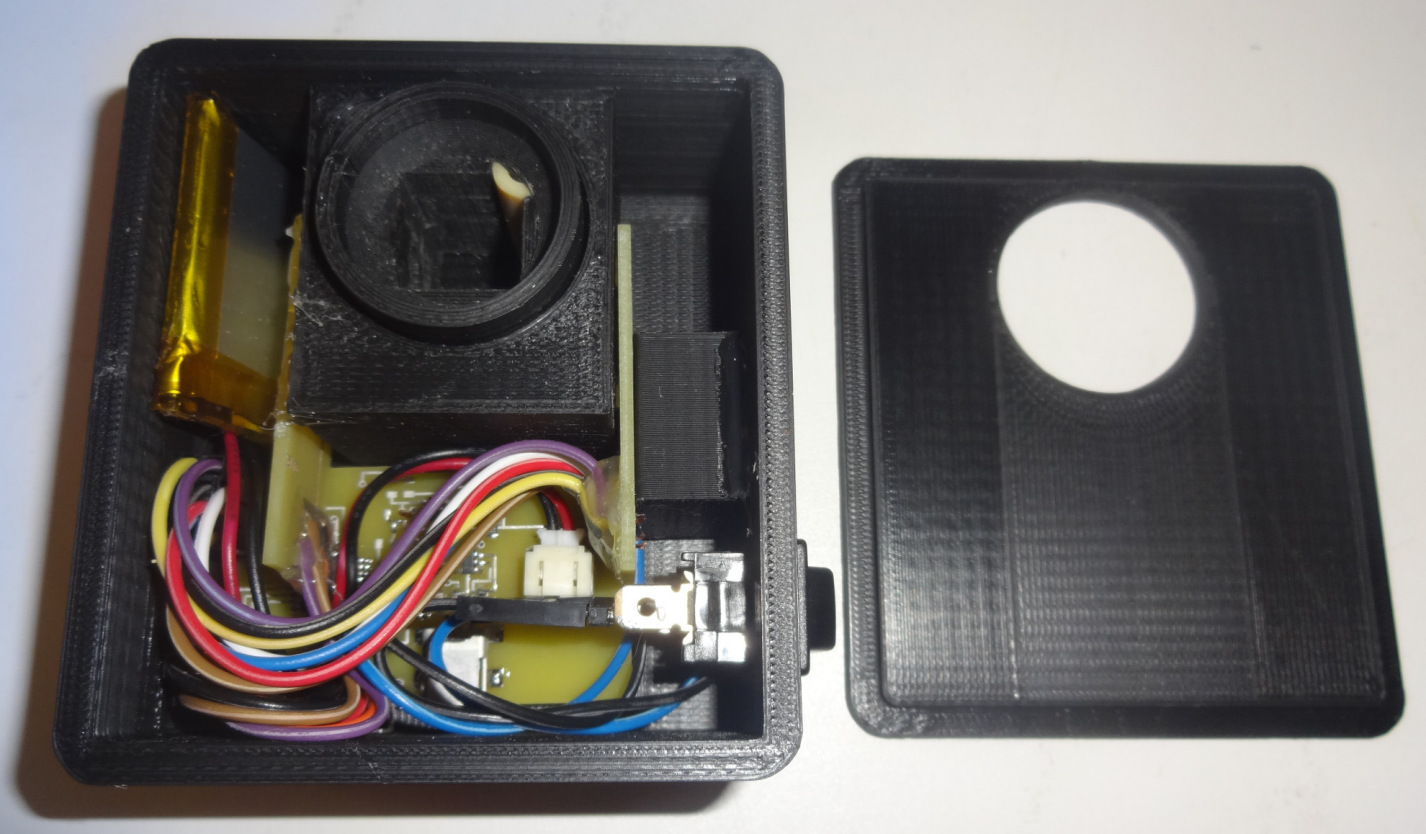
Photometer case with electronics inside.

Figs 4–7 show the final photometer case printed in black PLA to reduce stray light. There is a green cylindrical piece of TPE (printed in ninjaflex) to ensure a secure and consistent placement of the cuvette. Fig 6 also shows the optional TPE casing printed to give the case extra protection and shock absorption should it be dropped in the field.

Within the case are the control board, two sensor boards, power switch, battery, and all of the necessary wires. The case is designed to be as small as possible while allowing all items to be inside. Optimal wire lengths can be used to free up some space should a larger battery be desired.

**Fig 8:**
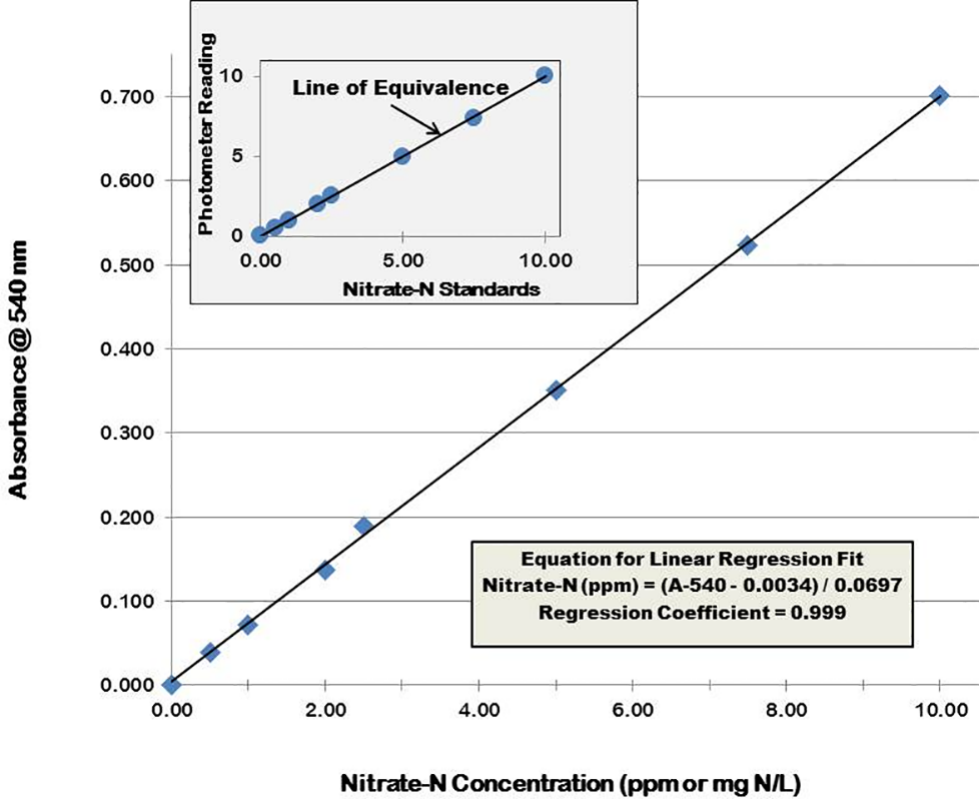
Nitrate Standard Curve used for calibration of the open-source photometer.

Fig 8 illustrates the relationship between concentration of nitrate-N (ppm) and the absorbance of the light within the sample when analyzed at 540 nm in an HP 8452a Diode Array Spectrophotometer. In this graphic, each point represents the mean of 5 independent standards for that nitrate concentration. The size of the symbol indicates the standard deviation of the mean for n = 5, which were all less than 10% relative standard deviation. A calibration curve for the sample is created from this data and its linear regression fit is programed into the software app for the photometer. The equation of the linear regression fitted line is shown in the box in Fig 8, which has a correlation coefficient = 0.999. The same nitrate standards were analyzed with the photometer and the inset graph in Fig 8 shows the results for the mean of 5 independent standards for each nitrate concentration plotted against the nominal concentration of the Nitrate standards. Again the size of the symbol shows the standard deviation of the photometer readings with none being greater than 10% of the mean. A line of equal equivalence is shown in the inset graph, where photometer reading would equal the nominal nitrate standard concentration.

This Standard Curve for Nitrate is incorporated in the app software and used to calculate the results displayed to the user in units of ppm nitrate-N. The user is also supplied with a 5 ppm nitrate-N standard which is processed via enzymatic reduction and reaction with the Greiss reagents. This standard permits the user to verify that the enzyme is working for nitrate reduction and the internal standard curve of the photometer is reporting correctly. Since the Enzymatic Nitrate Analysis Method reports combined Nitrate plus Nitrite in a sample, the user may also determine the nitrite content of a sample as described in the Methods section. In this case, the user would choose the EPA Nitrite option in the initial menu of the smartphone app and the results would be reported as ppm Nitrite-N. The user also has the option of obtaining the results as WHO units of ppm Nitrate and ppm Nitrite, when selecting at the initial menu. The ppm Nitrate is calculated by the app using a factor of 4.4 times the ppm Nitrate-N using the imbedded standard curve equation. For ppm Nitrite, the conversion factor is 3.3 times the ppm Nitrate-N. Nitrate and Nitrite content of water is evaluated in units of ppm Nitrate and ppm Nitrite, in California, European Union countries and the rest of the world. Only the US EPA uses units of ppm Nitrate-N and ppm Nitrite-N. It should be noted that nitrate-N is not a measure of total nitrogen.

**Fig 9:**
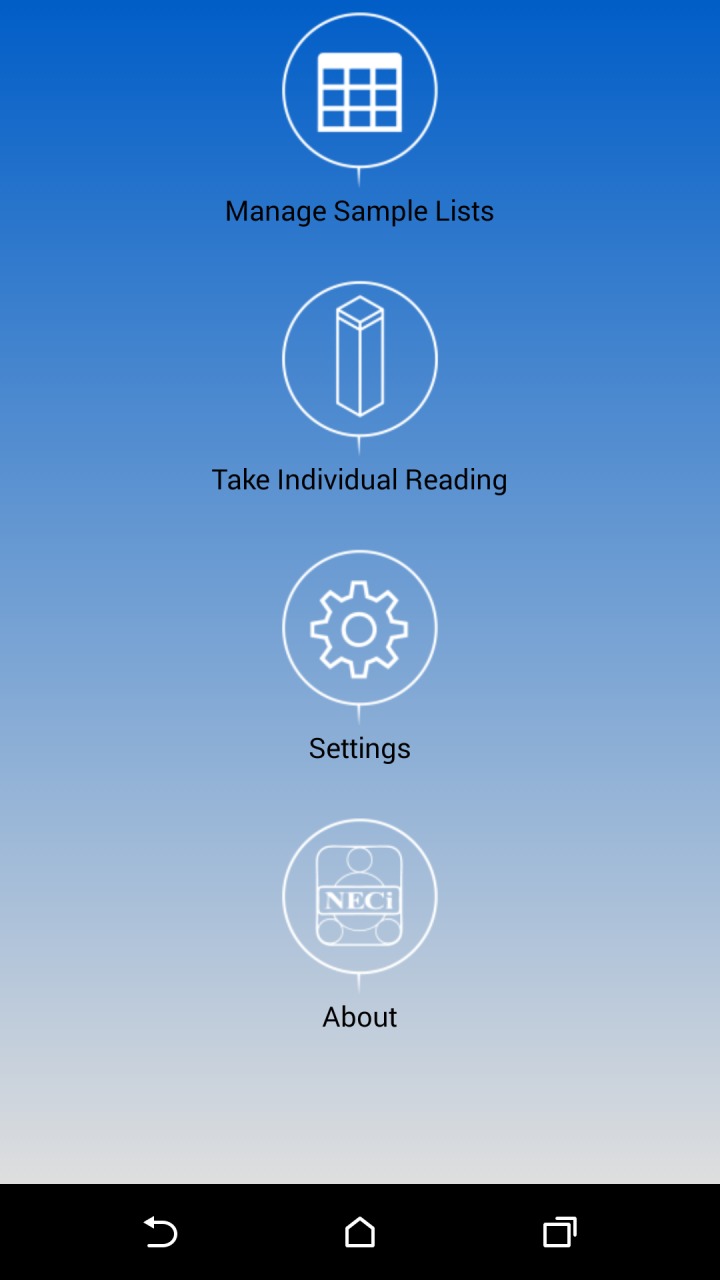
Screenshot of Android phone App interface for the Home Screen.

**Fig 10:**
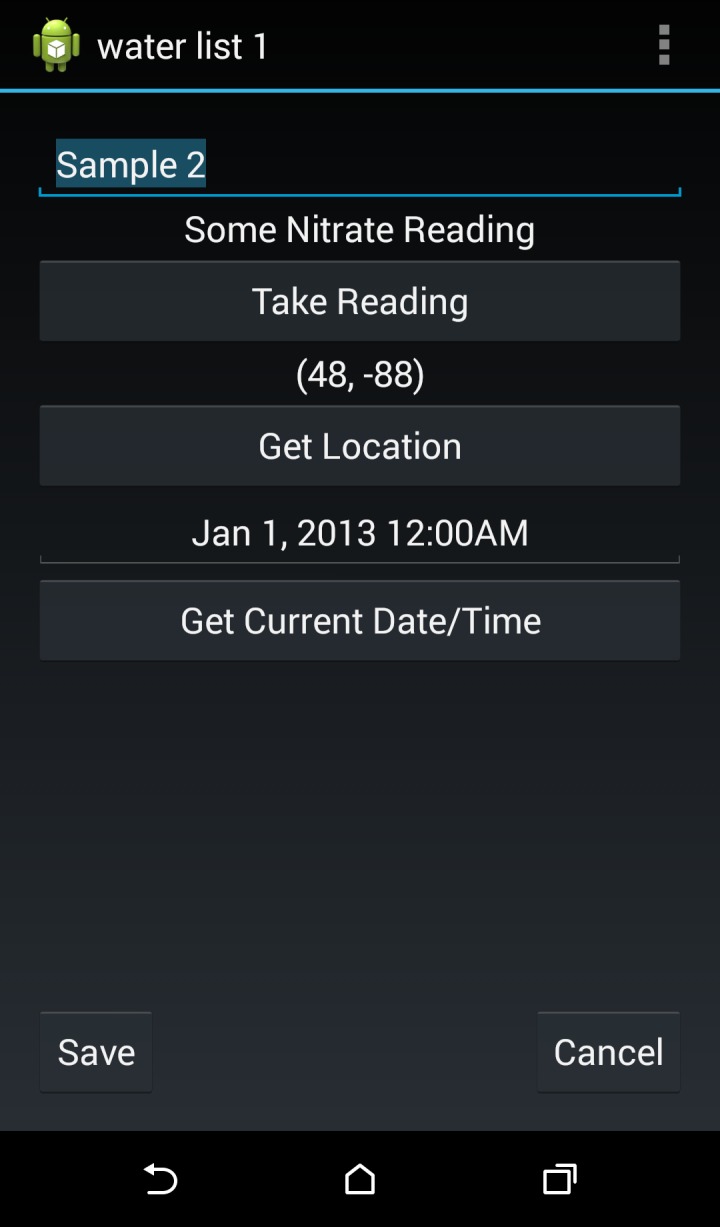
Screenshot of Android phone App interface for the options for a specific sample.

Screenshots for the open-source Android App interface are shown in Figs 9 and 10. The source code is available at https://github.com/NitrateEliminationCo/NECiOpenWater. From the home screen (Fig 9) users can 1) Manage saved samples from a sample list, 2) take a unique reading, 3) adjust the settings or 4) see the about screen with contact information for NECi. From the Managing Sample Lists users can create a new Sample List, save samples of that type or view and edit a list of saved samples. Once a Sample List is selected, the name of the list is displayed in the Action Bar as well as the ability to add a sample to the list and export the list to another application.

Table 1 shows the cost breakdown for the photometer case and electronics. After printing and assembly the photometer case consists of 74.68g of PLA and 29.90g of Ninjaflex TPE (30) for the rubberized outer casing.

**Table 1:** Cost breakdown of the photometer case and electronics (Digikey part # unless externally referenced).

Item	Qnty	Cost/qnty	Total Cost	Reference
PLA (kg)	0.07468	$24.95	$1.86	[39]
NinjaFlex (kg)	0.0299	$86.65	$2.59	[40]
RN-42 Bluetooth SPP Module	1	$18.32	$18.32	740-1038-ND [41]
Atmel Atmega328p (or 168p)	1	$3.41	$3.41	328-PU-ND
Power switch	1	$1.24	$1.24	450-1040-ND
3.7V 500mAh LiPo	1	$4.99	$4.99	[42]
361nm LED	2	$3.99	$7.98	[43]
Green LED	2	$0.50	$1.00	67-1121-ND
68 Ohm Current Limiting Resistor	5	$0.29	$1.45	RPC0805JT68R0CT-ND
Taos TSL238 Light-Frequency	4	$3.46	$13.84	TSL238-TCT-ND
10k Ohm Reset Pull-up	2	$0.10	$0.20	RNCP0805FTD10K0CT-ND
1uF Filter Capacitor	4	$0.31	$1.24	399-8001-1-ND
Bi-Color Red/Green LED	1	$0.50	$0.50	160-1058-ND
1k Ohm Pull-up Resistor	1	$0.10	$0.10	RMCF0402JT1K00CT-ND
USB mini-B Port	1	$1.07	$1.07	H2959CT-ND
USB Charge indicator 3mm LED	1	$0.50	$0.50	160-1058-ND
300 Ohm Current Limiting Resistor	1	$0.10	$0.10	311-300ARCT-ND
MAX1555 USB Charge Circuit	1	$2.03	$2.03	MAX1555EZK+TCT-ND
2 Pin JST Battery Connector	1	$0.56	$0.56	455-1749-1-ND
3.3V LDO	1	$0.61	$0.61	MCP1755T-3302E
		Grand Total	$63.59	

Currently, PLA can be purchased for $24.95 per kg [39] and NinjaFlex TPE [44] can be purchased for $64.99 per 0.75kg meaning the total material cost for the case of the photometer is just $4.45 [40]. Couple this cost for the case with the low cost of internals $59.14 mostly available at Digikey [45] and the total proposed cost, $63.59, for the open-source photometer is 14.32% of current retail options [46].

Nitrate testing using the enzyme nitrate reductase are not costly and favorably compare with commercial cost per test. In addition to the cost benefit of the new nitrate testing method, there are no harmful chemicals that are needed in the new nitrate testing platform. For details of the costs of nitrate test kits see [47,48] and for NECi Nitrate Test Kits, see nitrate.com.

## Discussion

Nitrate testing is needed for a number of applications including water testing, agricultural soil and plant testing, dry forage testing, and wastewater testing. Current nitrate testing platforms can cause hazards in the environment, are prohibitively expensive for all applications and are more challenging for users than necessary. For example, nitrate analysis normally involves reduction of nitrate to nitrite, that is then reacted with Griess reagents to yield a highly colored compound, which can be quantified with a colorimeter or spectrophotometer [11,12]. The reduction step in the reaction of the traditional methods uses copper-cadmium granules or hydrazine [49], both of which are toxic reagents.

Less expensive methods, such as nitrate test strips are unreliable. Different brands of test strips are used in a similar fashion: strips are briefly dipped into an extract solution (for soil) or in the test solution/water, and allowed to develop color during a standard interval of time (e.g. 30 and 60 seconds) and compared to a color chart, calibrated to either parts per million (ppm) of nitrate or expressed in ppm equivalents of nitrogen (NO3-N), is used to determine the nitrate concentration of the sample. In a comparison test by Murphy et al., no brand of test strip measured nitrate accurately below 10 ppm [50]. In addition, several brands of strips that measure nitrate in addition to other constituents in water were found to under estimate nitrate concentration, especially at high values [50]. There is, thus a need for a more accurate, safer, and less expensive complete methodology to test nitrate concentration in water and soil samples.

The Nitrate Elimination Co., Inc. (NECi) manufactures the recombinant enzyme, nitrate reductase, for use in nitrate testing [10]. The enzyme catalyzes the reduction of nitrate to nitrite in the presence of NADH, and replaces the toxic heavy metal cadmium or other toxic reagents used for this reaction. In both these methods, the nitrite produced from the reduction of nitrate and any nitrite in the sample reacts with the Griess reagents to produce a magenta color with absorbance at 540 nm. Thus, these methods report the nitrate plus nitrite content of a sample. For the nitrate reductase method, it is easy to determine the nitrite content of a sample by simply omitting the enzyme from the reaction and directly reacting with the Griess reagents to obtain absorbance at 540 nm.

Since nitrate reductase is a highly efficient catalyst, only a tiny amount is required for each reaction and the enzyme is very specific such that virtually no other substance is reduced in the reaction. The Enzymatic Nitrate Analysis Method is a green sustainable method, which is safe for the user and the environment. NECi packages nitrate reductase in a series of Nitrate Test Kits which cover the following applications: water testing, agricultural testing, seawater testing, and wastewater testing (see nitrate.com). These test kits are designed to provide all the tools that are needed to do nitrate analysis in the lab or the field and are relatively easy to use following simple instructions. For example, the test kits have been used in classrooms [51]. Recently, the U.S. EPA has proposed the Method for Nitrate Reductase Nitrate-Nitrogen Analysis (ATP Case No. N07-0003) as an alternative nitrate analysis method to existing certified EPA methods for nitrate analysis [13]. It has been recommended as an addition to EPA Approved Methods at 40 CFR Part 136. Thus, there is no longer a need for any nitrate analysis methods that require toxic reagents. With the addition of the open-source photometer, nitrate analysis can be done in the field with ease and precision.

Although the hardware for the nitrate testing photometer is provided in this study freely and is completely open-source the majority of the developed world market may still choose to purchase a factory assembled tool. However, providing the hardware designs freely has several important benefits as it provides additional value to companies supplying such open hardware, their customers, and the greater scientific community. First, just as FOSS is becoming the norm in software development [52–55], FOSH is establishing itself as a high-value method to enable companies to innovate quickly [56]. The potential result is an improved open-source spectrometer in the future with no costs associated with the research and development after the initial documentation. The use of distributed digital manufacturing techniques with 3-D printing for the manufacture of these cases is more cost effective for the individual or small or medium enterprise (SME) [57,58] and preliminary life cycle analysis also indicates it is better for the environment [59,60]. This and the open-sourcing the bill of materials (Table 1), the designs (Figs 1, 2 and S1 Appendix), and software allow for a greater number of people to use this technology and the new, safer nitrate testing technique. This greater access to the open source appropriate technology is most important in developing regions and for small farmers [61–66]. This greater access also creates the potential for increased customers for the materials that are not easily manufactured by the consumers such as the nitrate reductase. The coupling of materials manufacturers with open-hardware designing has been proposed before [67], but is still at an early stage of development.

As with any electronic device with a finite lifetime, when this device either wears out or is replaced by a newer better device extra waste is generated as old devices are replaced with new ones. However, the inherent modular nature of this open-source photometer developed here allow easy upgrades for new technology and testing methods allowing the physical device to last longer than a closed proprietary version. In addition, if a single component of the device fails (e.g. and LED or the cuvette holder) it can be easily replaced without the need to replace the entire device. Additionally the old parts may be disassembled more completely because of this modular nature allowing individual components to be recycled and reused where applicable instead of disposed of in a landfill. For example, when the cases are 3-D printed, recycling codes can be embedded in them [68] enabling waste pickers with access to the open-source recyclebot [69] technology to recycle the polymer back into fair trade 3-D printable filament [70]. This recycled filament both costs less than commercial filament, but also has environmental benefits as determined by life cycle analysis [59,71].

## Future Work

The modular and open hardware nature of the spectrometer encourages future development of the tool to increase applications and quality [17,56]. The low power consumption needed for the device position it very favorably to become completely solar powered removing the dependence on any established electrical grid system letting it be used in the most remote areas of the world. Better circuitry and electronics can yield a smaller device in future iterations with the ability for the device to become pocketable instead of handheld. With the current state of the device there is the potential for the photometer to grow with the rapid changing world of 3-D printing to the point where to entire device could be printed with the circuit boards, batteries, and even diodes. In addition, the spectrometer could be integrated more closely with the smart phone itself as there are open source smartphones under development such as the openmoko [72]. The capabilities of the device can also be expanded for other testing methods, such as for phosphate testing. This can then be better integrated with information for specific applications. For example, the GIS-tagged data can be integrated online and then be used to give feedback for users (e.g. farmers) such as recommendations on fertilizer use [73,74].

## Conclusions

The Nitrate Reductase Nitrate-Nitrogen Analysis Method allows for a complete elimination of toxic heavy metal for nitrate quantification as presented here creating a safer environment for all that use it. Additionally this method has been shown to be quicker, as accurate as and less expensive than commercial processes. Utilizing 3-D printing for the manufacture of the photometer allows the device to be user-upgraded and user-maintained. The open-source nature and concomitant reduced equipment costs creates the potential for a large network of users able to improve, fix, and re-distribute the design to reach the maximum audience possible. As shown in this study, the dramatic reduction of cost (85%) for the open-source photometer is significant enough and the price point (less than $65) is low enough where this technology can be obtained and used by people at nearly every income level.

## Supporting Information

S1 Appendix OpenSCAD design for photometer case. (TXT)
